# Impact of translation on named-entity recognition in radiology texts

**DOI:** 10.1093/database/bax064

**Published:** 2017-08-28

**Authors:** Luís Campos, Vasco Pedro, Francisco Couto

**Affiliations:** 1 LASIGE, Departamento de Informática, Faculdade de Ciências, Universidade de Lisboa, 1749-016 Lisboa, Portugal; 2 Unbabel Lda, Rua Visconde de Santarém, 67-B, 1000-286 Lisboa, Portugal

## Abstract

Radiology reports describe the results of radiography procedures and have the potential of being a useful source of information which can bring benefits to health care systems around the world. One way to automatically extract information from the reports is by using Text Mining tools. The problem is that these tools are mostly developed for English and reports are usually written in the native language of the radiologist, which is not necessarily English. This creates an obstacle to the sharing of Radiology information between different communities. This work explores the solution of translating the reports to English before applying the Text Mining tools, probing the question of what translation approach should be used. We created MRRAD (Multilingual Radiology Research Articles Dataset), a parallel corpus of Portuguese research articles related to Radiology and a number of alternative translations (human, automatic and semi-automatic) to English. This is a novel corpus which can be used to move forward the research on this topic. Using MRRAD we studied which kind of automatic or semi-automatic translation approach is more effective on the Named-entity recognition task of finding RadLex terms in the English version of the articles. Considering the terms extracted from human translations as our gold standard, we calculated how similar to this standard were the terms extracted using other translations. We found that a completely automatic translation approach using Google leads to F-scores (between 0.861 and 0.868, depending on the extraction approach) similar to the ones obtained through a more expensive semi-automatic translation approach using Unbabel (between 0.862 and 0.870). To better understand the results we also performed a qualitative analysis of the type of errors found in the automatic and semi-automatic translations.

**Database URL:** https://github.com/lasigeBioTM/MRRAD

## Introduction

Radiology reports describe the results of radiography procedures and have the potential of being an useful source of information (1), which can bring benefits to health care systems around the world. However, these reports are usually written in free-text and thus it is hard to automatically extract information from them. Nonetheless, the fact that most reports are now digitally available makes them amenable for using Text Mining tools. Another advantage of Radiology reports is that even if written in free-text, they are usually well structured. A lot of work has been done on Text Mining of Biomedical texts, including health records (2), but although Radiology reports are usually written in the native language of the radiologist, Text Mining tools are mostly developed for English. For example, Hassanpour *et al.* (3) created an information extraction system for English reports that depends on RadLex (4), a lexicon for Radiology terminology, which is freely available in English. Given this dependence, the system cannot be easily applied to reports written in other languages. And even if the system was not dependable on an English lexicon, it is not certain that the results would be the same if another language was used, because of, for example, differences in syntax [other examples of tools developed focused on English include (5–7)]. This has been an obstacle in the sharing of Radiology information between different communities, which is important to understand and effectively address health problems.

There are mainly two possible solutions to this problem. One is to translate the lexicon itself (8, 9) and the other is to translate the reports. Translating the lexicon has the advantage of not requiring continuous translation, i.e. after translating a lexicon to, e.g. Spanish, we can then use it to process as many untranslated Spanish reports as needed. However, when a new version of the lexicon is released the changes need also to be translated; otherwise, the translated lexicon would become outdated. Given the increasing evolution of translation services nowadays available, we decided to assess the alternative option of translating the reports and check its feasibility. This approach has the advantage that the translated reports would be accessible to any doctor who understands English. Consider the scenario of translating reports in a hospital in Portugal. This would benefit: (i) tourists that can access their reports in their language and send them to their personal doctor at home; (ii) the hospital that can get a highly specialized second opinion in complex clinical cases from international experts that do not understand Portuguese. Beyond that, any state-of-the-art Text Mining tools focused on English text could then be applied to the reports without any need for adaptation.

In both solutions, if the translation is done by experts on the field, we can probably assume that not much information is lost in translation. We call this type of translation Human Translation (HT). But expert translators are expensive, which makes this solution unscalable, with a high financial cost. Another option is to use Machine Translation (MT). Notwithstanding the lower translation quality, it is cheaper and scalable. Finally, an option that tries to get the best of both worlds is using MT with human Post-Editing (MT + PE). In this approach the text is automatically translated and then the translation is corrected by a human. Koponen (10) reviews the relevant literature and concludes that PE can be worth it, being cheaper than HT and with better quality than MT, but it depends on the quality of the MT. One approach that has been gaining traction is the use of the crowd to do the Post-Editing (11). The advantages of this strategy include lower per-word cost and sometimes an higher speed, compared with HT. One big disadvantage is less assurance of quality.

Thus in this work we aimed at addressing the following question: what kind of translation should be used to translate non-English Radiology reports to then apply Text Mining tools? To the best of our knowledge, currently there is no publicly available study that provided a quantitative evidence to answer this question. This could be explained by the lack of a parallel corpus that could be used to study this. The most similar work we discovered in the literature is Ref. (12). The author finds that a rule-based MT translation system has a good performance in translating medical text from Portuguese to English, for using an Information Extraction system. But no comparison between translation systems is done, as we provide in this work.

Specifically, we focused on the Text Mining task of Named-entity recognition (NER). This is a relevant task since the outputs from NER systems can be used in Image Retrieval (13) and Information Retrieval (14) systems and can be useful for improving automatic Question Answering (15). NER has the goal of locating and classifying all the Named-entities in a certain document. Named-entities are elements of the text that belong to one of certain predefined classes. For example, there are NER systems that can recognize mentions of chemical entities (16), diseases (17) or terms from specific ontologies like HPO (Human Phenotype Ontology) (18).

Considering all this, the main contributions of this article are:

- A Portuguese–English parallel corpus of research articles related to Radiology, called MRRAD (Multilingual Radiology Research Articles Dataset), containing for each article the original Portuguese document, the HT translation, two alternative MT translations and a MT + PE translation. The corpus if freely available online (https://github.com/lasigeBioTM/MRRAD).
- Measurement of the performance of multiple automatic or semi-automatic translation approaches in the task of translating Portuguese Radiology-related text to English, for the purposes of recognizing RadLex terms in the translated text (a lexicon-based NER task).

## Materials and Methods

### MRRAD corpus

To the best of our knowledge there is no parallel corpus of Radiology reports, so we created a Portuguese–English parallel corpus of research articles related to Radiology, assuming that the writing style and content of these research articles are similar to Radiology reports.

For each research article the MRRAD corpus contains the original Portuguese text, a HT translated English text, a MT translated English text using Yandex, a MT translated English text using Google (Statistical Approach, not the most recent Neural Approach) and a MT + PE translated English text using Unbabel.

To obtain a list of research articles related to Radiology, we used the NCBO Entrez Programming Utilities (E-utilities) (https://www.ncbi.nlm.nih.gov/books/NBK25501/) to query the PubMed database with the search query ‘portuguese[Language] AND english[Language] AND radiography[MeSH Major Topic] AND hasabstract[text]’ (search done on 11 December 2016). The last filter was used to avoid getting texts for which only the title was available. Then we programmatically crawled the PubMed page for each article to get the URL where the full article could be found. Most of the articles were hosted in SciELO (http://www.scielo.br/) and only articles hosted in there were included in the corpus. More, only articles for which the original language is Portuguese are included in the corpus. Finally, we programmatically crawled the SciELO pages for each article to get both the original Portuguese text and the English translation. From the HTML of each page we extracted everything from the abstract until, but not including, the references/bibliography.

Three of the articles were surveys, not containing much vocabulary about Radiology (PMIDs: 19936506, 22002140, 23515770). They were excluded from the corpus. Other two contained encoding problems and were also excluded (PMIDs: 21793046 and 24263777).

The final result is a parallel corpus of 51 articles, distributed by journal as shown in Table 1.

**Table 1.** Number of corpus articles per journal

Journal	Number of articles
Arquivos Brasileiros de Cardiologia	24
Jornal Brasileiro de Pneumologia	14
Revista do Colégio Brasileiro de Cirurgiões	4
Brazilian Journal of Otorhinolaryngology	2
Arquivos Brasileiros de Cirurgia Digestiva	2
Revista Brasileira de Cirurgia Cardiovascular	2
Jornal da Sociedade Brasileira de Fonoaudiologia	1
Einstein (São Paulo)	1
Revista Brasileira de Reumatologia	1

To give a sense of the corpus size, the human English translations have a total of 163 423 words [Tokenization done by NLTK’s *word_tokenize* function (http://www.nltk.org/)], the longer article having 12 451 and the smaller 848 words. The articles have an average of 3204 words each.

It is not known for sure how exactly the original HTs were performed, since some of the articles are not recent and some of the journals did not answer our request for more information about the translation, but all the answers received mentioned the use of specialized translation services. Having said this, it is being assumed that the translations are of high quality since they are published by scientific magazines.

We used Yandex’s free Translate API (https://tech.yandex.com/translate/) to machine translate the Portuguese version of the articles. Yandex is a Russian company which, among other things, sells automatic translation services, but it has a limited free service. It currently uses a Statistical approach to MT. Each translation request had a limit of 10 000 characters so we developed software to break the text to various pieces, without breaking the text in the middle of sentences, send the translation request for each piece and then join everything back.

Finally the English translations using Google and Unbabel were obtained with Unbabel’s API (http://developers.unbabel.com/). Unbabel is a Portuguese start-up which sells translation services, using a MT + PE approach. Here’s the simplified pipeline:

- Text is translated by MT (in this case, using Google Translate, Statistical Approach);
- Machine Translated text is Post-Edited by users of Unbabel’s platform. Users translate the text using Unbabel’s web-interface or mobile application; and
- Translation resulting from last step is reviewed by an Unbabel’s senior user, an user that was promoted for having good ratings;

We obtained Google’s Statistical MT using the *mt_translation* endpoint of the API and Unbabel’s MT + Post-Editing using the *translation* API’s endpoint. The requests for Unbabel Translations have a limit of words, so we used a software similar to the one utilized for the Yandex Translations.

### Annotations

We annotated all the English translations with the Open Biomedical Annotator (OBA) (19) using the REST API (http://data.bioontology.org/documentation\#nav_annotator). OBA is an open-source tool for NER, using a lexicon-based approach. It uses a case-insensitive direct match approach, not considering lexical variations of words. We are calling this annotation strategy Direct Match. We used the default API parameters, namely, the ones shown in Table 2.

**Table 2.** NCBO annotator parameters used

Parameter	Value
expand_class_hierarchy	false
expand_mappings	false
minimum_match_length	3
exclude_numbers	false
whole_word_only	true
exclude_synonyms	false
longest_only	false

To experiment with different matching strategies, we also annotated the articles using NOBLE Coder (http://noble-tools.dbmi.pitt.edu/) (20). Like OBA, it is also a lexicon-based system but unlike OBA, NOBLE can find mentions of lexical variations of the terms present in the lexicon provided. This tool was chosen against other similar tools because of its comparable quality and higher ease of use. We annotated each of the articles twice with this tool, using two different built-in matching strategies, Best Match and All Match. These strategies differ from one another and from Direct Match on how flexible they are in finding matches between terms in a lexicon and the words in a text. For example, the Best Match approach only matches the most specific term in the text, e.g. if the words *lung cancer* are present in the text, the Direct and All Match approaches will match both *lung* and *lung cancer*, while the Best Match approach will only match *lung cancer*. More information about NOBLE annotation strategies can be found at Ref. (20). We used the GUI interface to upload the RadLex ontology to NOBLE Coder and the command-line interface to obtain the annotations (See https://github.com/lasigeBioTM/MRRAD/blob/master/notes_on_dataset_creation/using_noble_coder.md for more information about how NOBLE Coder was used).

To note that the three of these annotation methodologies take into account not only the preferred name of a certain RadLex term but also its synonyms.

For each document and annotation approach we created the set of the RadLex terms (identified by their RIDs) that were found in that document with that annotation approach. This is the data used in the assessment of translation solutions that follows.

### Experimental setup

The RadLex terms extracted from each MT or MT + PE translated article were compared against the RadLex terms extracted from the corresponding HT translated article, which was considered a gold standard. Both Micro- and Macro- Precision, Recall and F-scores were calculated. This was done for each annotation approach.

$$\text{Precision}\;(P)=\frac{\text{TP}}{\text{TP}+\text{FP}}$$

$$\text{Recall}\;(R)=\frac{\text{TP}}{\text{TP}+\text{FN}}$$

$$F-\text{Sscore}=\frac{2.P.R}{P+R}$$

F-score is the harmonic mean of Precision and Recall. In these equations, TP is the number of true positives (number of RadLex terms extracted from the translation being evaluated that are also extracted from the HT), FP is the number of false positives (number of RadLex terms extracted from the translation being evaluated that are not extracted from the HT) and FN is the number of false negatives (number of RadLex terms not extracted from the translation being evaluated but that are extracted from the HT).

To facilitate the understanding of the results, we will now walk through a short example. Consider that we have one Portuguese document and corresponding HT English translation and MT English translation. Four terms of interest were identified in the HT translation, {bone, cell, finger, colon} (We use here human understandable names instead of RIDs so that the example is easier to follow). This is going to be our gold standard. In the MT translation, two terms of interest were found, {brain, bone}. One of these terms is also in the gold standard, which means TP = 1, but the other term is not, FP = 1. In the gold standard there are three terms that were not found in the MT translation, which means FN = 3. After calculations, this gives us a Precision score of 0.5, a Recall score of 0.25 and F-score of 0.33.

We calculated these scores using both Micro and Macro approaches. In the Micro approach, the TP, FP and FN values of each document are summed and then the Precision, Recall and F-score are calculated using the formulas exposed above. With this approach, we obtain the Micro Precision, Micro Recall and Micro F-score. In the Macro version the Precision, Recall and F-score for each document are calculated and then the results for all documents are arithmetically averaged. With this we obtain the Macro Precision, Macro Recall and Macro F-score. These methods measure how similar are the terms extracted from MT or MT + PE texts to the terms annotated on the HT texts.

## Results and Discussion

Table 3 presents the number of RadLex extracted by document using the different annotation approaches. One of the highlights here is that the All Match approach consistently found more terms than the Best Match approach, which itself found more terms than the Direct Match approach. This was expected since the All Match approach it’s the most flexible one in what it considers to be a mention of a RadLex term. The Best Match approach is more strict than the All Match approach but less than the Direct Match approach, considering lexical variations and word reordering, for example. But in all cases we can see that many terms are being extracted from each document.

**Table 3.** Number of RadLex terms found by document

Translation	Direct match	All match	Best match
Human	119.55	177.92	145.00
Yandex	116.06	173.92	145.16
Google	120.80	179.49	147.61
Unbabel	120.92	178.86	148.16

**Figure 1.**
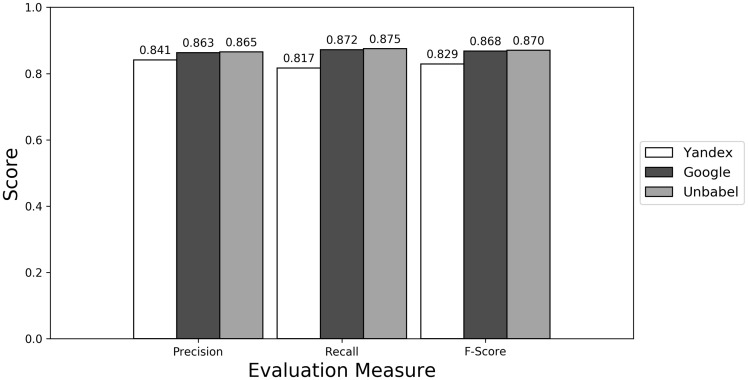
Micro evaluation of test translations (direct match).

**Figure 2.**
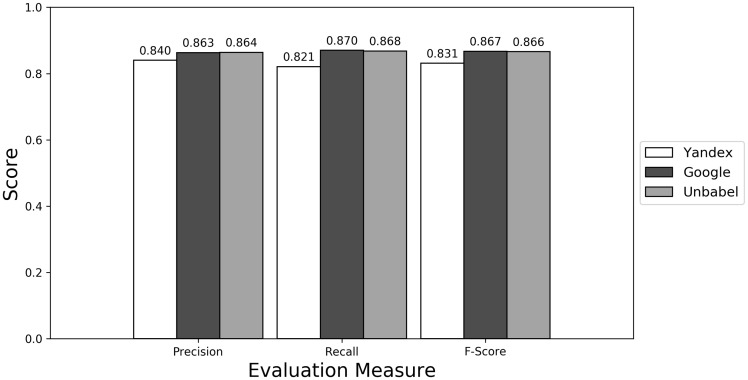
Micro evaluation of test translations (all match).

**Figure 3.**
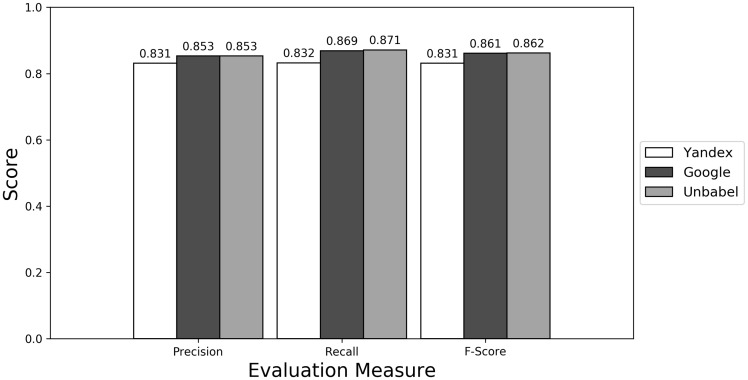
Micro evaluation of test translations (best match).

As seen in Figures 1–3 (Supplementary Table S1 presents the data in table format), the terms extracted from Google translations are more similar to the ones extracted from HT translations than the ones from Yandex translations. This could be just because the human translators used Google Translator to help them in their translation process. This argument loses strength if we assume that Google Translate translation outputs changed since the articles were human translated (publication years of the articles in MRRAD range from 2003 to 2013), but data could not be found to corroborate this assumption.

The terms extracted from Unbabel and Google translations are really similar, the F-scores being almost equal. That the translations are similar is not too surprising since the Post-Editing phase at Unbabel is done after MT translation using Google. What could be surprising is that Unbabel does not have a higher score. One conclusion to take from this is that Post-Editing step on the MT + PE does not add value for this task. The results are similar when a Macro Evaluation is done (see Supplementary Table S2).

So, for this task, if someone had to choose between Google and Unbabel, this someone would be better off using Google since it is cheaper. But one question remains, is it worth to use any of the machine or semi-MTs systems? The terms extracted from any of these translations are not extremely different but they are also not equivalent to the ones extracted from the HT. It could be the case that for some applications (like automatic Question Answering) only translations close to human quality are acceptable, while for other applications a mediocre translation would be good enough. Therefore, the suitability of the MT and MT + PE translations probably depends on the practical usage for these translations and annotations.

To better understand the results we will now provide a detailed analysis on the annotations for the ‘clinical finding’ and ‘anatomical entity’ subtrees of RadLex. These are two of the subtrees that probably would be more important when applying RadLex to a Information Retrieval system (21), a type of application for which the results of this study can be useful.

### Clinical finding and anatomical entity subtrees

**Figure 4.**
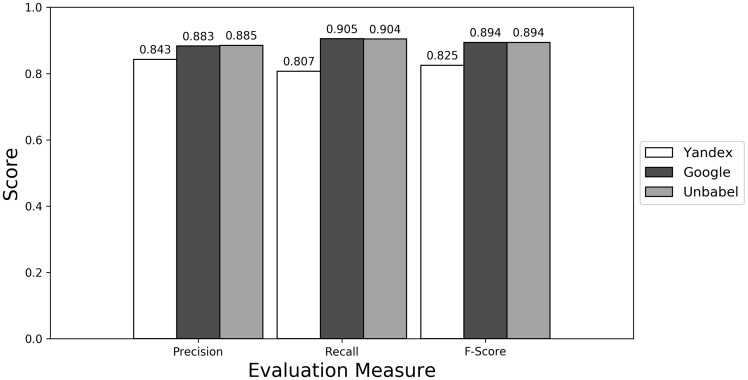
Micro evaluation of test translations considering just terms from RadLex ‘clinical finding’ and ‘anatomical entity’ subtrees (direct match).

**Figure 5.**
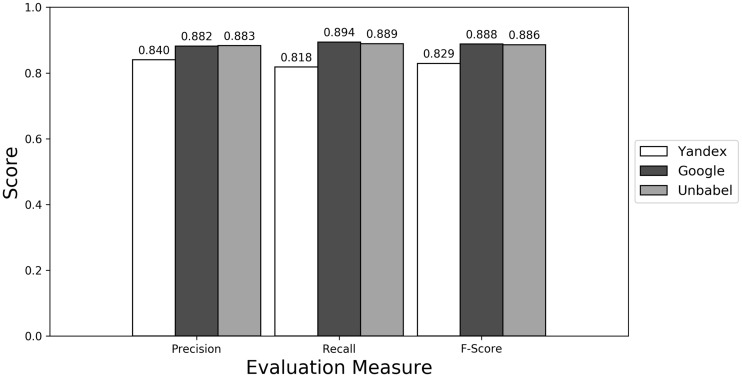
Micro evaluation of test translations considering just terms from RadLex ‘clinical finding’ and ‘anatomical entity’ subtrees (all match).

**Figure 6.**
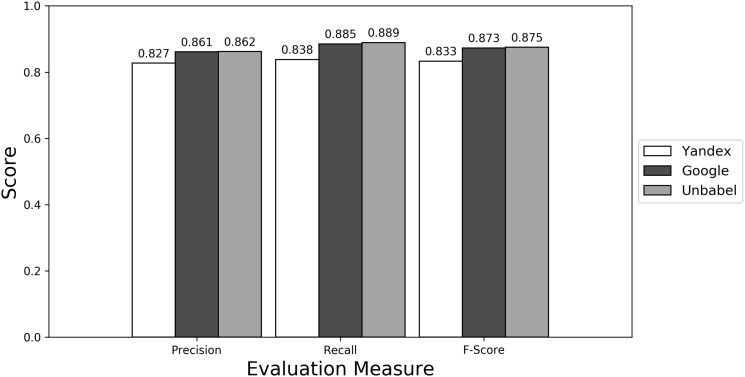
Micro evaluation of test translations considering just terms from RadLex ‘clinical finding’ and ‘anatomical entity’ subtrees (best match).

Depending on the type of annotation approach and translation it was found between 35.25 and 55.55 ‘clinical finding’ or ‘anatomical entity’ terms per document (see Supplementary Table S3). As seen in Figures 4–6 (see Supplementary Table S4 to see the data in table format), the scores obtained are similar to the ones obtained for all terms, with Yandex translation extracted terms being the less similar to the HT translation extracted terms and Google and Unbabel having similar scores. Similar results were found when Macro evaluation was performed (see Supplementary Table S5). But why these scores?

In an attempt to better understand the results, we have done an analysis of the False Positives and False Negatives errors committed by the MT and MT + PE translations, focusing on the terms belonging to the ‘clinical finding’ or ‘anatomical entity’ RadLex subtrees. From preliminary analysis we knew that some of the FPs and FNs are not caused by an erroneous translation but due to other causes, for example, an alternative translation which is correct but causes a different annotation, e.g. translating ‘parênquima pulmonar’ to ‘pulmonary parenchyma’ instead of to ‘lung parenchyma’. Both translations are correct but the second one leads to the extraction of the term ‘lung’ while the first does not. Still, we expected a higher number of real translation errors using Yandex compared with the Unbabel or Google translations, since both of these types of translation had better scores.

We done an analysis on the FPs and FNs errors committed by Yandex, Google and Unbabel translations in nine random documents and each error was classified by type (see Supplementary Table S6). The results from the Best Match Approach were used. As predicted, the percentage of errors of Yandex due to a wrong translation (25% of 100 FPs or FNs) was higher than the percentage of errors of Google and Unbabel (22.09% of 86 and 21.18% of 85 FPs or FNs, correspondingly), but only slightly (see Supplementary Tables S7 and S8). The reasons for the others FPs and FNs included, among others, cases (i) of different translations which are both correct but lead to different annotations, as described earlier and (ii) in which the word extracted does not have the same meaning in the text as it has in RadLex. For example, the case of extracting the anatomical term ‘hand’ from ‘(…) on the other hand, it has to be considered that (…)’, in which the word ‘hand’ is used metaphorically. This happens because a rule-based approach is being used, which does not consider the context of the term.

There were a lot of these (ii) cases, which maybe would happen less if a Machine Learning NER approach was used. We thought about this but the problem is that, to the best of our knowledge, there is no data readily available to conduct an experiment of this type, i.e. we could not find English Radiology text resulting from HT of Portuguese text and annotated with RadLex terms by experts.

Next we analyzed what kind of real translation errors were causing the FPs and FNs (see Supplementary Table S9). These subcategories included cases in which:

- There was an extra word in the translation;
- There was a missing word in the translation;
- A wrong hyphenation was used;
- An acronym was not translated;
- The test translation used a term that was too general;
- A wrong lexical variation was used; and
- The most correct medical term was not used;

Each of these cases had a really low number of occurrences and so it is not worth a deeper analysis. One interesting thing to note is that in the Yandex translations there were some cases (six) in which the original Portuguese word was not even translated. This never happened in the Google and Unbabel translations that were analyzed. This could be explained by the fact that probably Yandex focuses on different languages than Google and so their Portuguese-English translation and/or language models are not so well trained. But most of the errors correspond to just to a general wrong choice of terms to use as a translation. For example, translating ‘média’ to ‘middle’ instead of ‘mean’ or ‘lesões de via biliar’ to ‘lesions via bile’ instead of ‘lesions to the biliary tract’. This type of problems could probably be solved by training Google and Yandex models with more data, specifically data related to medicine.

One could expect that Unbabel translations would have a lot less mistakes than Google’s but this is not always the case. There are situations in which errors are even added during the Post-Editing step. A review of the errors makes us propose that this could be due to the lack of medical knowledge of Unbabel current editors. For example, a ‘stroke’ is something that occurs in the brain but in one case it was used as something that happens in the heart—someone with some knowledge on medicine would not make this error. But the truth is that Unbabel currently do not have a focus on medical content. We predict that if they did and invested in growing a crowd of experts with a better knowledge of medical language, this would lead to better results.

## Conclusions

In this article, we presented the MRRAD corpus, a corpus of 51 Portuguese research articles related to radiology and four alternative translations to English for each one of these articles. This corpus can be used to study the efficacy of translation solutions in biomedical text, particularly text related to Radiology. From the best of our knowledge this is the first corpus of this type. This corpus could even be extended by other researchers, using different types of translation or languages, for example.

Based on MRRAD, we also presented a quantitative evaluation of the performance of multiple automatic or semi-automatic translation approaches in the task of translating Portuguese Radiology-related text to English, for the purposes of recognizing RadLex terms in the translated text. To better understand the results we also did a qualitative analysis of the type of errors found. The results will certainly be helpful for the decision-making of developers who want to develop multilingual applications that apply Text Mining tools, specially in Radiology text. The results corroborates the conclusion that if the developers have limited financial resources to pay for HTs, using a MT service like Google is a better option than a service that implements Post-Editing, like Unbabel. Of course, maybe there are better MT services than Google or better MT + Post-Editing services than Unbabel is currently offering for the medical field, and this is something that could be explored in further work.

To note that, since the MRRAD articles are freely available online, there is the risk that the MT systems used were previously trained on these documents. If, for example, the Google MT system was trained on the documents but Yandex’s did not, there is going to be a bias in favor of Google’s system. We are not sure how big of an issue this is.

Regarding the fact that we just test commercial tools in this paper, we thought of using an open-source MT translation tool (e.g. Moses (http://www.statmt.org/moses/)) but this would involve training the system with training data. Since MRRAD corpus is not that big, we decided it was not worth it. Having said this, future work could involve repeating the experiment using a translation system trained just with text from the biomedical domain (22).

Since we are studying a way to annotate non-English text using English terms we think these results can motivate the sharing of annotations of biomedical text across communities. Linked-data (23) approaches, for example, will benefit from this sharing because they will have access to data that would be hard to access behind language barriers. This sharing will allow, for example, find reports from different languages when searching for Radiology reports about left shoulders.

During this work we only experimented with rule-based NER. The results could be different if some kind of Machine Learning based approach was used instead, something that could be explored in further work. More, here we just assessed the application of automatically recognize RadLex terms from translated text. A more realistic approach and a possible next step would be to test the performance of each kind of translation in a real application, like an Information Retrieval (24) or Question Answering system. But even if we discover which translation strategy is better for each kind of system, the question of the feasibility of integrating translation in systems used in real-word settings (e.g. hospitals) remains and this is something that could be explored in further work through, e.g. a partnership with a clinical facility. This is important since our evaluations were all done based on automatic procedures and it is not certain that the results would be the same if humans were involved in the evaluation process. Finally, we tested the solution of translating the reports, but since it also sounds like a viable option, the translation of lexicons is another option that should be experimented with in further work.

## Supplementary data

Supplementary data are available at *Database* Online.

## Supplementary Material

Supplementary Data
